# Comparison of characteristics and outcomes of patients admitted to hospital with COVID-19 during wave 1 and wave 2 of the current pandemic

**DOI:** 10.1007/s11739-021-02842-5

**Published:** 2021-10-12

**Authors:** David Fluck, Suzanne Rankin, Andrea Lewis, Jonathan Robin, Jacqui Rees, Jo Finch, Yvonne Jones, Gareth Jones, Kevin Kelly, Paul Murray, Michael Wood, Christopher Henry Fry, Thang Sieu Han

**Affiliations:** 1 Department of Cardiology, Ashford and St Peter’s Hospitals NHS Foundation Trust, Guildford Road, Chertsey, Surrey, KT16 0PZ UK; 2 Department of Acute Medicine, Ashford and St Peter’s Hospitals NHS Foundation Trust, Guildford Road, Chertsey, Surrey, KT16 0PZ UK; 3 Department of Quality, Ashford and St Peter’s Hospitals NHS Foundation Trust, Guildford Road, Chertsey, Surrey, KT16 0PZ UK; 4 Department of Respiratory Medicine, Ashford and St Peter’s Hospitals NHS Foundation Trust, Guildford Road, Chertsey, Surrey, KT16 0PZ UK; 5 School of Physiology, Pharmacology and Neuroscience, University of Bristol, Bristol, BS8 1TD UK; 6 Department of Endocrinology, Ashford and St Peter’s Hospitals NHS Foundation Trust, Guildford Road, Chertsey, Surrey, KT16 0PZ UK; 7 Institute of Cardiovascular Research, Royal Holloway, University of London, Egham, Surrey, TW20 0EX UK

**Keywords:** Mortality, Frequent readmission, Length of stay, Coronavirus, LACE index

## Abstract

In this study of patients admitted with COVID-19, we examined differences between the two waves in patient characteristics and outcomes. Data were collected from the first COVID-19 admission to the end of study (01/03/2020–31/03/2021). Data were adjusted for age and sex and presented as odds ratios (OR) with 95% confidence intervals (CI). Among 12,471 admissions, 1452 (11.6%) patients were diagnosed with COVID-19. On admission, the mean (± SD) age of patients with other causes was 68.3 years (± 19.8) and those with COVID-19 in wave 1 was 69.4 years (± 18.0) and wave 2 was 66.2 years (± 18.4). Corresponding ages at discharge were 67.5 years (± 19.7), 63.9 years (± 18.0) and 62.4 years (± 18.0). The highest proportion of total admissions was among the oldest group (≥ 80 years) in wave 1 (35.0%). When compared with patients admitted with other causes, those admitted with COVID-19 in wave 1 and in wave 2 were more frequent in the 40–59 year band: 20.8, 24.6 and 30.0%; consisted of more male patients: 47.5, 57.6 and 58.8%; and a high LACE (*L*ength of stay, *A*cuity of admission, *C*omorbidity and *E*mergency department visits) index (score ≥ 10): 39.4, 61.3 and 50.3%. Compared to wave-2 patients, those admitted in wave 1 had greater risk of death in hospital: OR = 1.58 (1.18–2.12) and within 30 days of discharge: OR = 2.91 (1.40–6.04). Survivors of COVID-19 in wave 1 stayed longer in hospital (median = 6.5 days; interquartile range = 2.9–12.0) as compared to survivors from wave 2 (4.5 days; interquartile range = 1.9–8.7). Patient characteristics differed significantly between the two waves of COVID-19 pandemic. There was an improvement in outcomes in wave 2, including shorter length of stay in hospital and reduction of mortality.

**Supplementary Information:**

The online version contains supplementary material available at 10.1007/s11739-021-02842-5.

## Introduction

The coronavirus disease (COVID-19) pandemic has emerged as the biggest cause of mortality world-wide in the past year [1]. Factors associated with increased risk of death from COVID-19 include older age [2, 3], male sex [3, 4], obesity [5], chronic illness [3, 5], low income and education, unmarried and immigrants from a low- or middle-income country [4]. However, there are other reasons that may also contribute to the excess mortality. During this COVID-19 pandemic, there were two distinct waves observed in almost every country [6]. Because of its novelty, there was limited experience in managing COVID-19 patients in the first wave. By the second wave, more evidence on the effectiveness of many therapies emerged, followed by the roll-out of new and effective vaccines. There was a significant reduction in mortality rates from COVID-19 in wave 2 in most countries [7]. Although rapid advances in treatment played a crucial role in the improvement of survival [8–12], there were many other measures recommended by public health bodies including: protection (shielding) of vulnerable groups, particularly older individuals and those living in residential care homes; greater hygiene control and social distancing; rapid testing systems; and the timing of lockdowns [13]. On the other hand, COVID-19 continues to evolve with new strains emerging globally, including in the UK. Consequently, or otherwise, a significant shift in patient demographics occurred between the two COVID-19 waves.

Hitherto, most studies have focussed on patient characteristics and mortality in wave 1, whilst information on changes of these factors with wave 2 is lacking. In this study of patients admitted with COVID-19 during the first 13 months of the pandemic, we examined differences between wave 1 and wave 2 in patient characteristics, including: age, sex and LACE index (an indicator of health status); outcomes in hospital including length of stay (LOS) and in-patient mortality; and post-discharge outcomes including early readmissions and short-term mortality. Patients admitted with other causes (non-COVID-19) during the pre-pandemic year were included for reference.

## Methods

### Study design, participants and setting

We analysed prospectively collected data of consecutive unplanned admissions to a single NHS hospital from 1st April 2019 to 31st March 2021, including the first case of COVID-19 admission on the 1st March 2020 to the endpoint of the study on the 31st March 2021. The data comprised mortality and clinical characteristics, as well as care quality, including: the LOS; readmission frequency; comorbidities; and the number of previous emergency department visits [14, 15].

### Measurement

Morbidities were coded according to the international classification of diseases (ICD-11) [16]. Information on unplanned admissions and frequency of readmissions within 28 days and mortality within 30 days of discharge from hospital was documented. The LACE index was computed from *L*ength of stay (score range 0–7), *A*cuity of admission (score 0 or 3), *C*omorbidity (score range 0–5) and *E*mergency department visits (score range 0 or 4)—these scores were summated to a scale of between 0 and 19 [17]. The cause of death after discharge was certified by the general practitioner who then notified our Medical Records department for documentation.

### Categorisation of variables

The pre-pandemic period was from 1st of April 2019 to 29th February 2020 and the pandemic period was from 1st of March 2020 to 31st of March 2021. In general medical admissions, a LACE index score ≥ 10 has been shown to associate with increased risk of adverse outcomes such as frequent readmissions and mortality [14]. Frequency of early readmissions were categorised either into a single readmission or ≥ 2 readmissions within 28 days of discharge.

### Statistical analysis

Chi-square tests were used to determine categorical variables including age bands, sex, LACE index and mortality in relation to COVID-19 wave 1 and wave 2. Kruskal–Wallis tests were used to assess non-normally distributed data sets (LOS) and logistic regression was used to assess the differences in COVID-19 wave 1 and wave 2 (predictor variables) in relation to mortality in hospital and within 30 days of hospital discharge (dependent variables). The data are presented as two models; model 1: unadjusted and model 2: adjusted for age and sex. Odds ratios (OR) are given with 95% confidence intervals (CI). Analyses were performed using IBM SPSS Statistics, v23.0 (IBM Corp., Armonk, NY).

## Results

### General description

A total of 10 173 patients were admitted in the pre-pandemic period: 47.7% men, 52.3% women; aged 18–107 years (mean = 68.3 years, SD = 20.0). In the pandemic period there were 12 471 patients: 48.7% men, 51.3% women; aged 18–105 years (mean = 68.1 years, SD = 19.5). Amongst patients admitted during the pandemic period, there were 11.6% (*n* = 1452) who presented with COVID-19, most of whom (90.2%) were diagnosed by PCR tests (ICD code: U07.1) and the remaining by clinical presentation (9.5%, ICD code: U07.2) or 0.3% (4 patients) on the basis of personal history (ICD code: U07.3 and U07.4) [16]. Amongst patients with COVID-19, there were more men (58.5%) than women (41.5%) (*χ*^2^ = 63.1, *P* < 0.001). Overall, all-cause mortality rates were 7.6% in hospital and 4.6% within 30 days of hospital discharge in the pre-pandemic period; corresponding figures were 8.2% and 3.9% in the pandemic period. Amongst COVID-19 patients, there were no group differences between patients with a positive PCR test and those with clinical diagnosis for mortality in hospital, 24.7% vs 22.5% (*χ*^2^ = 0.3, *P* = 0.321); within 30 days of hospital discharge, 2.8% vs 3.6% (*χ*^2^ = 0.2, *P* = 0.403). A high LACE index (score ≥ 10) was recorded in 40.8% of patients. There were no differences in the proportion of admissions to the intensive care unit between wave 1 and wave 2: 2.9% *vs* 2.4% (*P* = 0.832). A single early readmission within 28 days of discharge occurred in 7.9% and ≥ 2 readmissions occurred in 2.6% of all COVID-19 patients.

### Management of COVID-19

All patients were managed with supportive therapies including oxygen, anticoagulation, continuous positive airway pressure ventilation where necessary and physiotherapy. There were additional therapies introduced during wave 2 of the pandemic, including; antiviral medication (remdesivir) and high-dose steroids (dexamethasone). An expansion of COVID-19 dedicated wards and intensive care units was also developed. This Trust also created an innovative discharge pathway, the REspiratory Emergency Department (REED) clinic to assess patients referred for COVID-19 to prevent admission and reduce early readmission and mortality. Patients were discharged with a pulse oximeter and information pack and reviewed on days 1, 3 and 7 post-discharge. The video consultation software ‘Attend Anywhere’ was used alongside telephone consultations.

### Characteristics of COVID-19 wave 1 and wave 2, with reference to pre-pandemic period

**Fig. 1:**
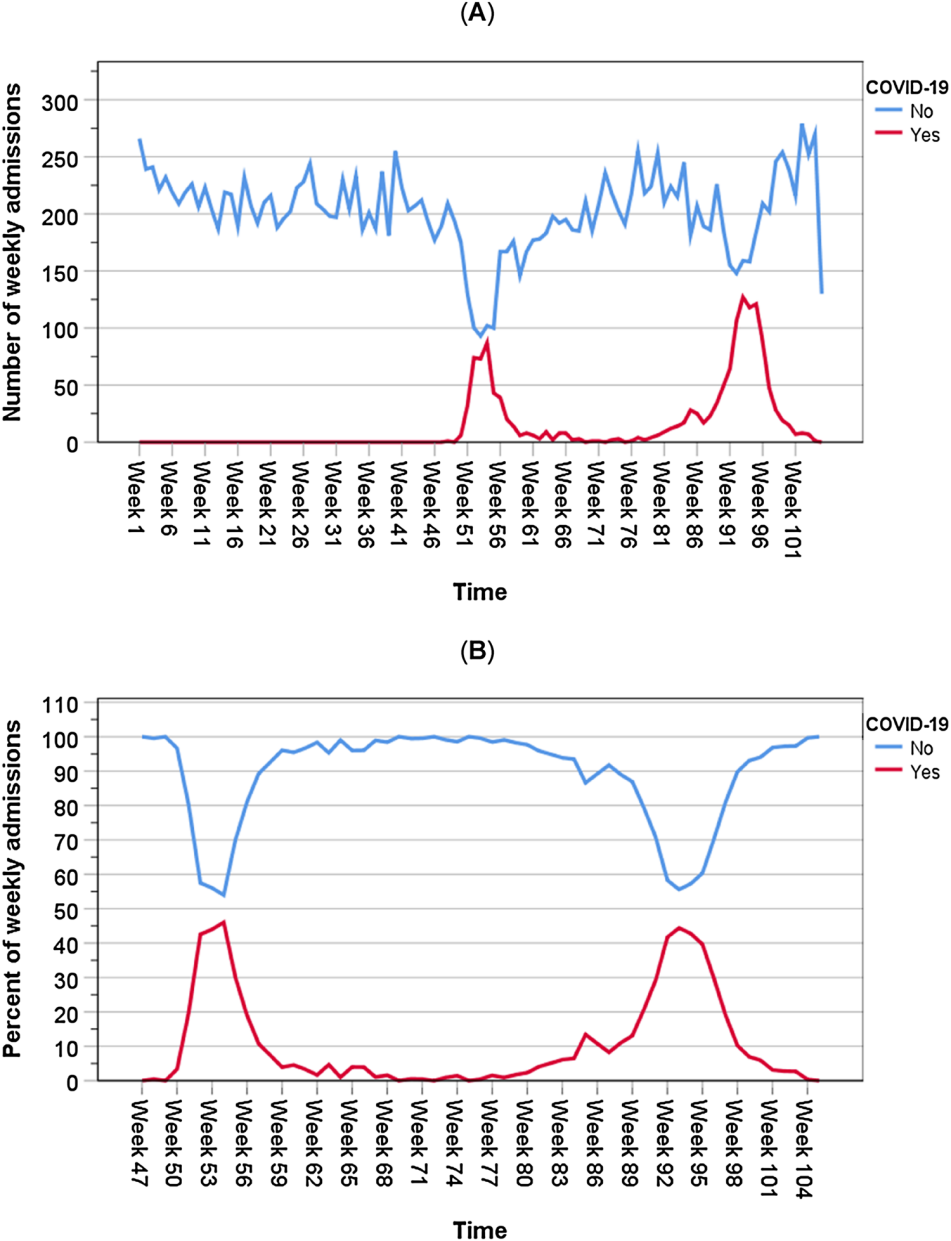
Numbers (**A**) and proportions (**B**) of admissions with COVID-19 and other causes between 01/03/2020 and 31/03/2021

There were two distinct waves of COVID-19: wave 1 from 01/03/2020 to 30/08/2020 and wave 2 from 10/09/2020 to 17/02/2021. Both wave 1 and wave 2 peaked at about 45% of all admissions in April 2020 and in January 2021, respectively. The initial slope in wave 2 was gradual and reached a small peak. By December 2020, the slope in wave 2 accelerated to its peak a month later (Fig. 1). On admission, the mean (± SD) age of patients in the pre-pandemic period was 68.3 years (± 20.0) and during the pandemic period was also 68.3 years (± 19.8); those with COVID-19 in wave 1 this was 69.4 years (± 18.0) and with COVID-19 in wave 2 was 66.2 years (± 18.4). The highest proportion of total admissions was seen among the oldest group (≥ 80 years) in wave 1 (35.0%). When compared with patients admitted in the pre-pandemic period, those admitted during the pandemic period with COVID-19 in wave 1 and in wave 2 were more frequent in the 40–59 year band (19.6, 24.6 and 30.0%); consisted of more male patients: 47.7, 57.6 and 58.8% and more had LACE indices ≥ 10: 38.8, 61.3 and 50.3% (Table 1).

**Table 1:** Patient characteristics according to COVID-19 status

	Pre-pandemic period		Pandemic period						Group differences	
	All causes (*n* = 10,173)		Other causes (*n* = 11,019)		COVID-19 wave 1 (*n* = 349)		COVID-19 wave 2 (*n* = 1103)		*χ*^2^	*P*
	*n*	%	*n*	%	*n*	%	*n*	%		
Age on admission (yr)										
18–39	1205	11.8	1188	10.8	22	6.3	101	9.2	87	< 0.001
40–59	1989	19.6	2292	20.8	86	24.6	331	30.0		
60–79	3342	32.9	3708	33.7	119	34.1	350	31.7		
≥ 80	3637	35.8	3831	34.8	122	35.0	321	29.1		
Sex										
Men	4854	47.7	5229	47.5	201	57.6	649	58.8	66	< 0.001
Women	5319	52.3	5790	52.5	148	42.4	454	41.2		
LACE index ≥ 10		38.8		39.4		61.3		50.3	123	< 0.001
ICD code										
U07.1	–	–	–	–	349	100	961	87.1	50	< 0.001
U07.2	–	–	–	–	0	0	142	12.9^†^		
Mortality										
In hospital	772	7.6	862	6.0	114	32.7	242	21.9	648	< 0.001
Within 30 days of discharge	436	4.6	419	4.0	14	6.0	18	2.1	16.1	0.005
Readmissions										
Single readmission	936	9.2	853	7.7	32	9.2	105	9.5	20.0	0.003
≥ 2 readmissions	299	2.9	281	2.6	9	2.6	29	2.6		

Admission, sex and mortality data as % of column totals

*ICD code: diagnosed on the basis of positive anti-COVID antibodies (ICD code U07.1) and clinical presentation (ICD code U07.2). ^†^Including four patients with diagnosis based on history (one (U07.3 and three U07.4)

Mortality rates in hospital and after discharge were consistently highest among patients admitted with COVID-19 in wave 1: the respective mortality rates in hospital within groups of patients in the pre-pandemic and in the pandemic with COVID-19 in wave 1 and with COVID-19 in wave 2 were 7.6% (*n* = 772), 32.7% (*n* = 114) and 21.9% (*n* = 242) and the corresponding post-discharge figures within 30 days were 4.6, 6.0 and 2.1%. This percentage for COVID-19 patients in wave 1 was 27.5% (114/414) and in wave 2 was 58.5% (242/414), i.e*.* proportionally more patients died after discharge in wave 1 than in wave 2 (*χ*^2^ = 7.1, *P* = 0.007). There were no group differences in readmission rates (Table 1).

The ages of death in hospital from COVID-19 in wave 1 (80.9 ± 11.3 years) and in COVID-19 wave 2 (79.6 ± 12.7 years) did not differ from that in patients admitted in the pre-pandemic (81.9 ± 111.8 years) and pandemic with other causes (81.1 ± 12.4 years) (ANOVA: *P* = 0.075). Survivors were significantly younger among wave 1 (63.9 ± 18.0 years) and wave 2 (62.4 ± 18.0 years) than those in pre-pandemic (67.2 ± 20.2 years) and pandemic with other causes (67.5 ± 19.7 years) (ANOVA: *P* < 0.001). Wave-2 patients were 1.5 years (− 4.3 to 1.4) younger than those in wave 1.

**Fig. 2:**
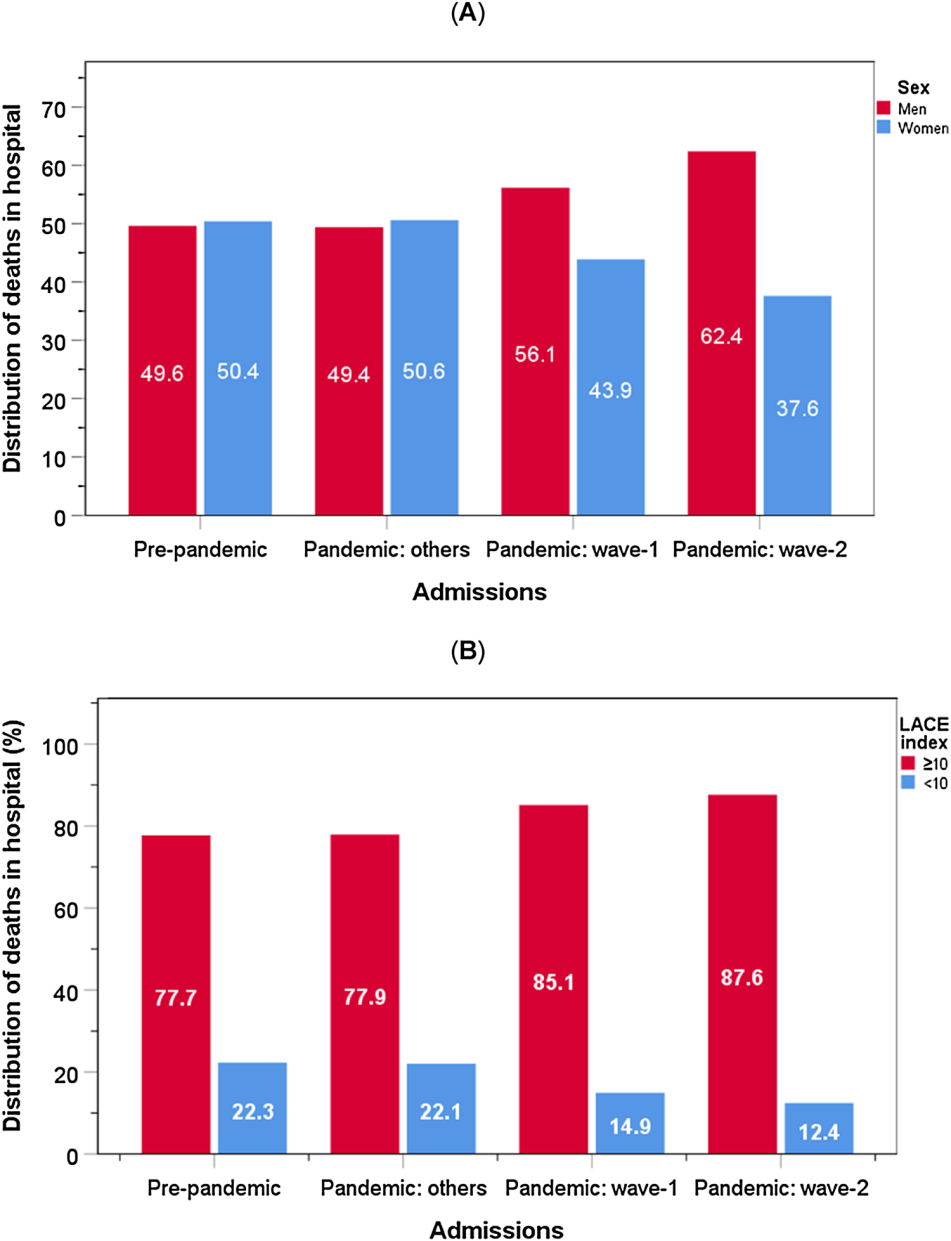
Distribution of deaths in hospital amongst men and women (**A**) and those with a high LACE index (**B**) by admissions in pre-pandemic period and COVID-19 pandemic period

There was a higher male to female patient percentage who died in hospital from COVID-19 in wave 1 (56.1: 43.9%) and wave 2 (62.4: 37.6%) when compared with that from other causes in the pre-pandemic (49.6: 50.4%) and pandemic (49.4: 50.6%) periods; *χ*^2^ = 14.7, *P* = 0.001 (Fig. 2A). There were more patients who died in hospital with a high LACE index (score ≥ 10) than those with an index < 10 amongst patients in all four groups, those admitted in the pre-pandemic period (77.7% vs 22.3%); the pandemic period from non-COVID-19 causes (77.9% vs 22.1%) and those admitted with COVID-19 in wave 1 (85.1% vs 14.9%) or in wave 2 (87.6 vs 12.4%); *χ*^2^ = 14.7, *P* = 0.001 (Fig. 2B).

### Length of stay in hospital

**Fig. 3:**
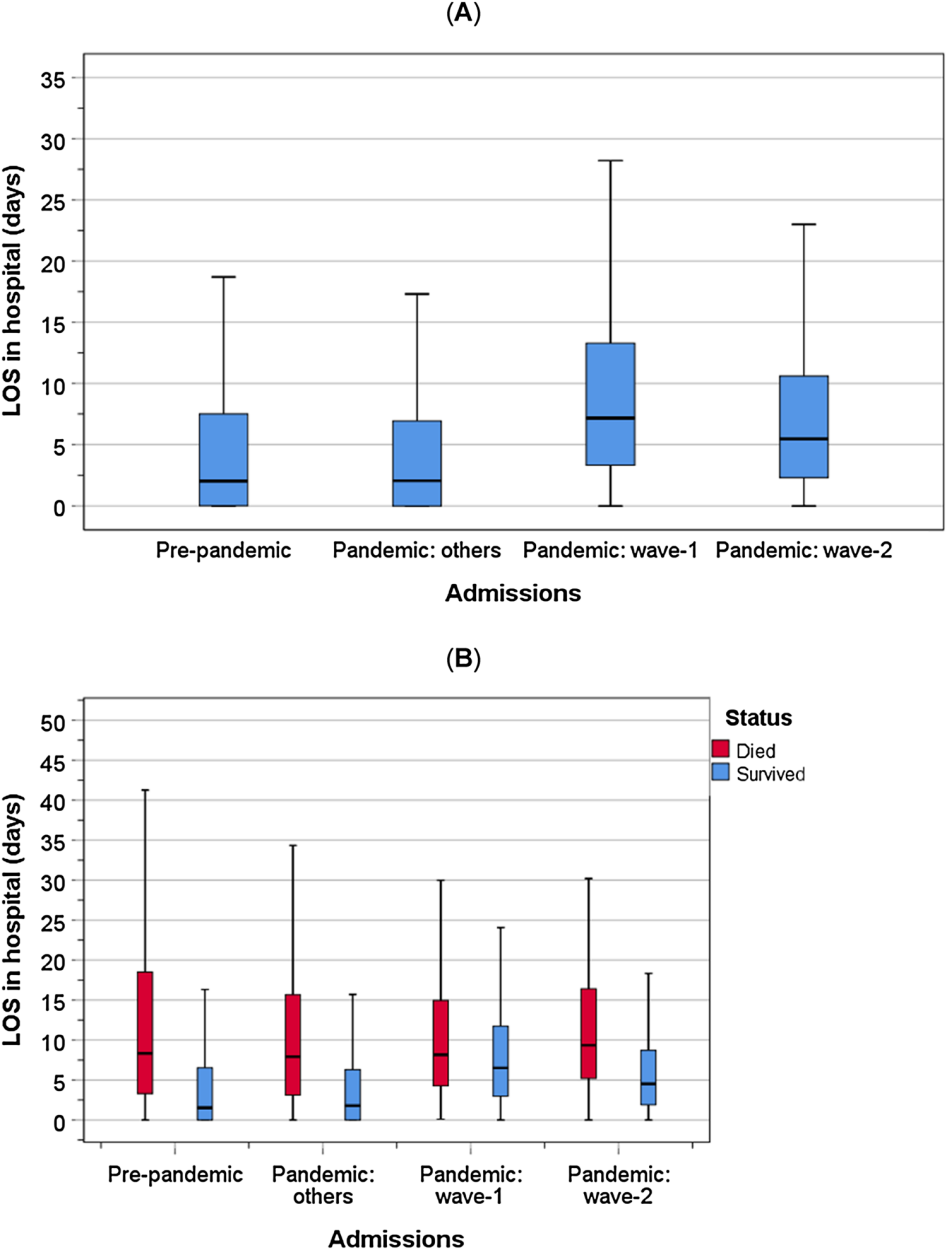
Length of stay in hospital amongst patients who died in hospital and those who survived to discharge. Bars represent the 5th and 95th percentiles

The LOS differed between groups of admission (Kruskal–Wallis test: *χ*^2^ = 482, *P* < 0.001). Patients admitted with COVID-19 in wave 1 stayed the longest in hospital (median = 7.2 days, IQR = 3.3–13.4), followed by wave 2 (median = 5.5 days, IQR = 2.3–10.6), compared with the shortest LOS among non-COVID-19 patients admitted in the pre-pandemic (median = 2.0 days, IQR = 0–7.5) and the pandemic (median = 2.0 days, IQR = 0–6.9) periods (Fig. 3A).

Separate analysis stratified by survival status showed that the median (IQR) LOS in hospital among those who died in hospital were similar for those admitted in the pre-pandemic (8.3 days; 3.3–118.5) and in the pandemic with other causes (7.9 days; 3.1–15.7) and with COVID-19 in wave 1 (8.2 days; 4.3–15.0) and in wave 2 (9.4 days; 5.2–16.5) (Kruskal–Wallis test: *χ*^2^ = 308, *P* < 0.001). In contrast, survivors of COVID-19 in wave 1 spent a longer time in hospital (median = 6.5 days; 2.9–12.0) compared to survivors of COVID-19 in wave 2 (4.5 days; 1.9–8.7) and to survivors of non-COVID-19 related causes in the pre-pandemic (1.5 days; 0–6.5) and pandemic periods (1.8 days; 0–6.3) (Kruskal–Wallis test: *χ*^2^ = 10, *P* = 0.019) (Fig. 3B).

### Mortality

Logistic regression showed that compared to patients admitted in the pre-pandemic period, those admitted with COVID in wave 1 had greater age- and sex-adjusted risk of death in hospital: OR = 6.87 (95%CI = 5.33–8.87) and death within 30 days of discharge: OR = 1.63 (95%CI = 0.93–2.85). Patients admitted with COVID-19 in wave 2 also had significantly greater risk of death in hospital: OR = 4.35 (95%CI = 3.66–5.16), but lower risk of death within 30 days of discharge from hospital: OR = 0.57 (95%CI = 0.35–0.92). The risk of death in hospital and within 30 days of hospital discharge among those admitted with other causes during the pandemic was lower than that in the pre-pandemic period (Table 2).

**Table 2:** Risk of death among patients admitted with COVID-19 wave 1 and wave 2 as compared to those who were admitted with non-COVID-19 related causes in the pre-pandemic and pandemic periods

	Logistic regression					
	Model 1: unadjusted			Model 2: adjusted for age and sex		
	OR	95%CI	*P*	OR	95%CI	*P*
In-patient mortality						
Pre-pandemic period (reference)	1	–	–	1	–	–
Pandemic period: other causes	0.78	0.70–0.87	< 0.001	0.78	0.70–0.87	< 0.001
Pandemic period: wave 1	5.91	4.67–7.48	< 0.001	6.87	5.33–8.87	< 0.001
Pandemic period: wave 2	3.42	2.92–4.02	< 0.001	4.35	3.66–5.16	< 0.001
Mortality within 30 days						
Pre-pandemic period (reference)	1	–	–	1	–	–
Pandemic period: other causes	0.87	0.76–0.99	0.041	0.86	0.75–0.99	0.040
Pandemic period: wave 1	1.30	0.75–2.26	0.345	1.63	0.93–2.85	0.090
Pandemic period: wave 2	0.44	0.27–0.71	0.001	0.57	0.35–0.92	0.021

When compared with the wave-2 group (reference), those admitted in wave 1 had significantly greater risk of death in hospital: OR = 1.58 (95%CI = 1.18–2.12), death within 30 days of discharge: OR = 2.91 (95%CI = 1.40–6.04) (Table 3). Further adjustment for the LACE index altered the associations between these variables slightly, but the same conclusions were drawn.

**Table 3:** Comparison of risk of death within 30 days of discharge between patients admitted with COVID-19 in wave 1 and wave 2 of the pandemic

	Logistic regression					
	Model 1: unadjusted			Model 2: adjusted for age and sex		
	OR	95%CI	*P*	OR	95%CI	*P*
In-patient mortality						
Wave 2 (reference)	1	–	–	1	–	–
Wave 1	1.73	1.32–2.25	< 0.001	1.58	1.18–2.12	< 0.001
Mortality within 30 days						
Wave 2 (reference)	1	–	–	1	–	–
Wave 1	2.97	1.45–6.06	< 0.001	2.91	1.40–6.04	< 0.001

## Discussion

The present study covered 13 months since the first case of COVID-19 was admitted to our centre. We observed distinct differences in characteristics and outcomes between wave 1 and wave 2 of the pandemic, both of which also differed from those who were admitted with other (non-COVID-19) causes and those admitted in the year before the pandemic. Overall, there was an improvement in outcomes for patients admitted in the second wave of COVID-19, as compared to the first, including better survival rates and shorter LOS in hospital. These observations provide valuable insights into the progress in managing this novel disease. It is likely that patient characteristics will continue to evolve with changes to the environment and management, particularly as new variants of COVID-19 continue to emerge in different parts of the world and while a number of new drugs and vaccines are made available for combating this disease [1].

### Observation 1: differences in patient characteristics between the two waves

The trends of both pandemic waves observed in our study synchronised with those of national data [18]. Compared to COVID-19 wave 2, there were fewer admissions during wave 1. This difference was likely due to higher community levels of COVID-19 underlying wave 2. The later acceleration of admissions in wave 2 was possibly due to the emergence of the alpha (Kent) variant. Although we did not have information to ascertain the variants of COVID-19, the trends in wave 2 of our centre mirrored the spread of the alpha variant which originated in the neighbouring county of Kent [19]. Other factors that might have influenced the rate of admissions in wave 2 included the expanded “NHS Test and Trace” programme [20]. This led to more cases being identified early, including those without symptoms, while the threshold of COVID-19 severity for admission also was somewhat lowered in wave 2. In this study, we only focussed on patients admitted to hospital therefore no information on symptom severity before hospital admission was available. In a separate, unpublished study of the same population, we found that amongst those who died of COVID-19, there was a significantly higher proportion of patients who presented with pneumonia (diagnosed by chest X-ray or computerised tomography scan) in wave 2 than in wave 1: 63.1% versus 45.6%, *χ*^2^ = 16.1, *P* < 0.001).

Among patients admitted in wave 2, their ages at admission were lower than those of patients in wave 1, which is likely related to changes in the at-risk population due to the high death rates among older and poorer health in wave 1, as well as greater protection strategies in the community as the pandemic progressed. Furthermore, it is possible that changes in community referral behaviour or physician admission behaviour could also have impacted on these differences.

There was only a slightly higher proportion of men than women admitted in wave 2 as compared to wave 1, but there were relatively higher rates of mortality among men in both waves of COVID-19. The reasons for this male preponderance of mortality remain unclear, since men were younger than women (67.4 years vs 68.8 years, *P* < 0.001) and had similar proportions of high LACE index (40.6% vs 41.3%, *P* = 0.466). The higher rate of male mortality observed in our study is similar to previous reports on other respiratory viruses such as influenza [21]. The high proportion of high LACE index scores (≥ 10) amongst patients with COVID-19 suggests that individuals with poorer underlying health were more susceptible to contracting COVID-19 and at greater risk of death. During wave 1, 60.3% of patients admitted with COVID-19 had a high index LACE index, which reduced to 50.3% in wave 2. Frailty as judged by the LACE index might have been segregated with age or again be influenced by mortality profile in wave 1. More aggressive shielding of vulnerable adults with underlying health conditions in wave 2 may explain this reduction. As far as we are aware, no previous studies have related the LACE index (a measure of underlying health status) to COVID-19 outcomes.

### Observation 2: in-hospital outcomes

The major observations were: LOS prior to discharge (longer in wave 1); length of stay prior to death (shorter in wave 1); and in hospital mortality (higher in wave 1). Almost certainly the competing interactions between the intrinsic risk of the cohort and the effectiveness of hospital care underpin this observation. The differences are very significant and it is likely that clinical intervention improvements and experience gained in wave 1 contributed to better outcomes in wave 2. The two cohorts had a very different risk profile based on age and frailty measures (LACE index), however the adjusted risk of in-hospital death was greatly different, again suggesting an intervention effect. The longer in-patient stay until death seen in wave 2 may reflect intervention strategies that will have improved overall survival, but also delayed time to death.

### Observation 3: post-hospital outcomes

Post-discharge mortality was high in wave 1, while wave 2 was much lower, even in comparison to standard general medical admissions in the pre-pandemic period. Competing interactions between the intrinsic risk of the cohort and the effectiveness of hospital care underpin this observation. It is likely that treatment of COVID-19 had improved over time, which enabled patients to recover faster and be discharged safely. This includes the much greater use of home oxygen during wave 2 at our centre. Our findings are consistent with those observed in the UK and most countries [7]. Age may also partly explain these differences since survivors to discharge was slightly younger in wave 2 than in wave-1 patients (1.5 years). In addition, fewer survivors of wave 2 had LACE index ≥ 10 (38.5%% vs 45.7%, *P* = 0.035). It was also reassuring that there were no differences in the frequency of early readmission.

By contrast, other regions have reported higher mortality rates in their second waves, including some Central and Eastern European countries [22], India, South Africa, Brazil and Mexico [6]. A number of factors may explain these increases including the lack of testing capacities, more lax preventative measures including social distancing and lockdown not strictly or impossibly imposed [23–25], overwhelmed healthcare systems and inadequate supply of medications and vaccines. The emergence of new coronavirus strains also plays a major role in countries such as India, South Africa and Brazil [26].

### Implications of changes in management of COVID-19 on clinical outcomes

As the pandemic progressed, improvement in the management of COVID-19 was likely to be an influential factor determining the outcomes observed in this study. As evidence of their positive benefit emerged from a number clinical trials [27, 28], the broad-spectrum antiviral agent remdesivir and dexamethasone were introduced from the later stages of wave 1 to treat more severe cases with COVID-19 disease [29–31]. Furthermore, experience gained from wave 1 was valuable for subsequent management of COVID-19 in wave 2. In addition, there was an increase in hospital capacity by the expansion of COVID-19 wards and intensive care units that reduced the risk of overwhelming capacity with COVID-19 admissions (as observed by a more gradual rise of admissions in wave 2). The REED clinic, designed to support management of COVID-19 in the community, has shown promising results in preventing hospital admission (unpublished data). We have also observed that amongst those who died of COVID-19, there were proportionally more patients who had multiple ward moves in wave 1 than in wave 2 (Supplementary Fig. 1), which might have compromised continuity of care and increased the risk of adverse outcomes.

Overall, our observations showed a number differences between the two waves of the COVID-19 pandemic, which were mostly consistent with other studies of European populations. This included findings that in wave 2: COVID-19 patients were younger [32, 33] and with fewer co-morbidities [33] and suffered lower rates of mortality [7, 34]. This study also contributed to the hitherto very limited data on differences in hospital LOS and post-hospital mortality between the two waves. Furthermore, the uses of pre-pandemic data as a control group and of the LACE index as a maker of underlying health status is unique. We believe that our findings provide valuable experience to the development of ongoing care regimes for patients with COVID-19.

### Strengths and limitations

The strengths of this study lie in its large number of consecutively admitted patients, covering more than a complete year of study since the first admission of COVID-19. Also the data were obtained from a single centre which minimised differences in care and intervention measures over the study period that otherwise might have acted as confounders if several centres were involved. On the other hand, because this was a single-centre study, our findings should be interpreted with this caveat since management of COVID-19 may have differed to other centres, particularly in other countries. The wide range of variables including LOS and the validated LACE index during hospital admission and after hospital discharge (early death and readmission) provides an in-depth explanation of some underlying reasons for differences between the two waves of COVID-19. It is possible that patients’ anthropometric characteristics, such as body mass index, may have differed between the two waves; however, this information was not collected. We did not collect information on the community COVID profiles comparing wave 1 and wave 2 or admission from care homes (one of the most vulnerable groups of individuals). However, the LACE index was available which is highly correlated with underlying ill health. We did not record nosocomial COVID-19 (but this would be low) or discharge destination and palliation.

In conclusion, patient characteristics differed between the two waves of COVID-19 pandemic. There was an improvement in outcomes in wave 2, including shorter LOS in hospital and a reduction in mortality.

## Supplementary Information

Below is the link to the electronic supplementary material.

Supplementary file1 (DOCX 85 kb)

## Abbreviations

CI: Confidence interval
COVID-19: Coronavirus disease-19
ICD: International classification of diseases
IQR: Interquartile range
LACE: Length of stay, acuity of admission, comorbidity and emergency department visits
LOS: Length of stay NHS National Health Service
OR: Odds ratio
REED: REspiratory Emergency Department
SD: Standard deviation
