# Supplementary material for: Comparison of characteristics and outcomes of patients admitted to hospital with COVID-19 during wave 1 and wave 2 of the current pandemic
Source: Intern Emerg Med. 2021 Oct 12;17(3):675–84. doi: 10.1007/s11739-021-02842-5 (PMC8505475; doi:10.1007/s11739-021-02842-5)

**SUPPLEMENTARY MATERIAL**

**Figure 1.** Mortality rates according to the number of ward moves in wave-1 and wave-2 of COVID-19 pandemic.


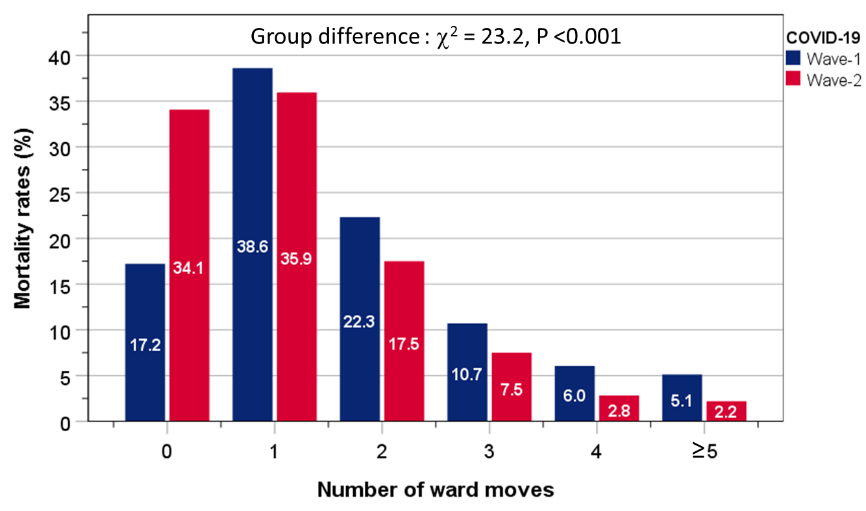

Supplement: Supplementary file 1 — Supplementary file1 (DOCX 85 kb) [file 11739_2021_2842_MOESM1_ESM.docx]
